# Alerting, Orienting, and Executive Control: The Effect of Bilingualism and Age on the Subcomponents of Attention

**DOI:** 10.3389/fneur.2019.01122

**Published:** 2019-10-30

**Authors:** Tanya Dash, Pierre Berroir, Yves Joanette, Ana Inés Ansaldo

**Affiliations:** ^1^Centre de recherche de l'Institut Universitaire de Gériatrie de Montréal, Montreal, QC, Canada; ^2^École d'orthophonie et d'audiologie, Faculté de médecine, Université de Montréal, Montreal, QC, Canada; ^3^Institute of Biomedical Engineering, Department of Pharmacology and Physiology, Faculty of Medicine, Université de Montréal, Montreal, QC, Canada

**Keywords:** bilingualism, subcomponents of attention, neuroimaging, attention network task, aging

## Abstract

Life-long experience of using two or more languages has been shown to enhance cognitive control abilities in young and elderly bilinguals in comparison to their monolingual peers. This advantage has been found to be larger in older adults in comparison to younger adults, suggesting that bilingualism provides advantages in cognitive control abilities. However, studies showing this effect have used a variety of tasks (Simon Task, Stroop task, Flanker Task), each measuring different subcomponents of attention and raising mixed results. At the same time, attention is not a unitary function but comprises of subcomponents which can be distinctively addressed within the Attention Network Test (ANT) (1, 2). The purpose of this work was to examine the neurofunctional correlates of the subcomponents of attention in healthy young and elderly bilinguals taking into account the L2 age of acquisition, language usage, and proficiency. Participants performed an fMRI version of the ANT task, and speed, accuracy, and BOLD data were collected. As expected, results show slower overall response times with increasing age. The ability to take advantage of the warning cues also decreased with age, resulting in reduced alerting and orienting abilities in older adults. fMRI results showed an increase in neurofunctional activity in the frontal and parietal areas in elderly bilinguals when compared to young bilinguals. Furthermore, higher L2 proficiency correlated negatively with activation in frontal area, and that faster RTs correlated negatively with activation in frontal and parietal areas. Such a correlation, especially with L2 proficiency was not present in young bilinguals and provides evidence for a bilingual advantage in the alerting subcomponent of attention that characterizes elderly bilinguals' performance. This study thus provides extra details about the bilingual advantage in the subcomponent of attention, in older bilinguals. Consequently, speaking more than one language impacts cognition and the brain later in life.

## Introduction

Enjoying a satisfying aging, particularly with regard to cognitive health, is desirable by people globally. Not all individuals enjoy healthy cognitive functioning, and even those who do usually show structural changes in their brain with aging. The mismatch between the relative preservation of cognitive abilities in the presence of structural changes in the brain is conceived as if the brain had some sort of “cognitive reserve,” a heavily used term defined as the individual differences in cognitive task performances which provides resilience to age-related brain damage or pathology (3, 4), giving rise to disparity between the degree of brain damage or pathology and the clinical manifestation of cognitive performance. Cognitive reserve is usually estimated using different intercorrelated factors—education (5), occupation (6), and second language learning (7–9). These factors have been studied in literature over the years as proxies of cognitive reserve.

In the context of lifelong bilingualism, cognitive reserve can be conceptualized as a cognitive resilience resulting from the use of, and exposure to, two or more languages. In such a context, it is believed that practiced bilingualism provides cognitive resilience to the cognitive control mechanisms allowing to counter, partially or totally the impact of age-related changes in the brain. Various studies suggest that lifelong bilingualism, or speaking two languages on a daily basis, can result in advantage in cognitive control processes i.e., how individual with high or low level of bilingualism or when compared to their monolingual peers differ in their behavioral (7, 10–12) and brain functions (11, 13–17). For example, it has been reported that elderly bilinguals show faster response time and make fewer errors than their monolingual peers on attentional control tasks (7, 18). Over the years, neuroimaging studies have also shown neural efficiency for bilingual elderly groups when compared to monolingual counterparts suggesting less activity in the prefrontal cortex (PFC) and anterior cingulate cortex (ACC) (11, 13, 16). Interestingly, 13 showed a comparable neurofunctional activation pattern for elderly bilinguals when compared to young adults. However, they outperformed monolinguals peers while showing less activation in frontal regions in a task-switching paradigm. Thus, suggesting neuroprotective effects of bilingualism in older adults on cognitive control processes. Furthermore, such a bilingual advantage in older adults is seen as a different strategy in cognitive control mechanism i.e., in the proactive mode of cognitive control (16). In proactive mode of cognitive control, attention is recruited as an “early selection” mechanism that relies on anticipation and prevention of interference before it occurs (19). In addition, functional connectivity studies have also supported better neural efficiency in bilinguals by demonstrating stronger resting state functional connectivity between anterior to posterior brain areas (14) and default mode network (DMN), and fronto-parietal cortex (17) for bilinguals compared to monolingual older adults. Berroir et al. (15) also suggest efficient performance for the bilingual older adults in the task-based functional connectivity measures.

At the same time, cognitive control is not a single mechanism (selection, inhibition, interference, switching, etc.) and it is thought to operate via the attentional functions (19) in a goal-directed manner. In fact, attention consists of subcomponent processes that are separable, yet interconnected which determines the order of the information processing (2, 20). These subcomponents of attention—alerting, orienting and executive control—can be measured by using the Attention Network Test (ANT, 1) whose validity has been proven across a variety of populations (21, 22). The neurofunctional bases of the three subcomponents of attention are themselves different from each other (1). Alerting function has been associated with various frontal and parietal regions with strong thalamic involvement. Orienting network has been associated with parietal sites and frontal eye field. Anterior cingulate cortex (ACC) as well as dorsolateral prefrontal cortex (DLPFC) are associated with execution network (1, 22). Very few studies have looked at the role of bilingualism on the subcomponents of attention (12, 23, 24). Moreover, these studies essentially focused on a behavioral comparison with monolingual individuals, suggesting enhanced alerting (12) and executive control (12, 23, 24) with no difference in orienting ability in bilinguals. And none of these studies compared the performance within bilingual groups varying in L2 age of acquisition (early vs. late), usage (balanced vs. unbalanced) or proficiency (high vs. low). There are studies that support the role of bilingualism on cognitive performance by performing correlation analysis of the measures of bilingualism (L2 usage and proficiency, age of acquisition) (25) with cognitive task (26, 27) instead of comparing dichotomous groups (like monolingual vs. bilingual; High proficient vs. low proficient bilinguals). In a study by Tse and Altarriba (26), more proficient individuals were better at maintaining attention during the task and these results were supporting the bilingual advantage. In addition, Luk et al. (27) also reported a positive correlation between age of onset of active bilingualism and flanker effect, suggesting that early bilingual experience resulted in greater advantage in cognitive control performance. Given that bilingual experience is dynamic in nature, the idea of treating bilingualism as a continuous variable is important (25, 28, 29).

In addition, age-related changes in elderly population—without measures of bilingualism—show significant reduction in the alerting (30, 31) and executive control abilities (31) when compared to young adults. However, the behavioral and neurofunctional bases of bilingual advantages in subcomponents of attention in aging remain unknown. In the present study, it is proposed to use the functional magnetic resonance imaging (fMRI) and the attention network task to understand the role of bilingualism while controlling for education—other proxies of cognitive reserve—to better understand the dynamic nature of interaction in aging population. The bilingual advantage conferred by lifelong use of two languages in aging may be associated with improved behavioral and neural efficiency. The goal of this study is thus to determine whether elderly bilinguals' show a behavioral and neurofunctional advantage over young adults—matched on measures of bilingualism as well as education—in different subcomponents of attention as measured by the ANT task. It is expected that older bilinguals will show longer response time and lesser accuracy, in the ANT performance. This will be reflected by an increase in the executive control effect and decrease in alerting and orienting effects in older bilinguals. In terms of fMRI data, we expect more neurofunctional activation in the older bilinguals when compared to young bilinguals in the executive control and alerting abilities (30, 31). More specifically, we expect that older bilinguals will recruit more fronto-parietal areas and anterior cingulate cortex when compared to young bilinguals for the ANT task performance. For the bilingual advantage hypothesis, we expect the negative correlations between L2 measures (age, usage, and proficiency), and ANT behavioral performance and BOLD activation for the subcomponents of attention.

## Methods

### Participants

Thirty-eight French-English bilingual young adults (YA; mean age 32.6 ± 3.1 years; *N* = 20; 9 females) and old adults (OA; mean age 73.94 ± 2.8 years; *N* = 18; 11 females) with no history of neurological or psychiatric disease were included in the study. A signed informed consent approved by the CRIUGM was obtained from each participant before the experiment.

### Tasks

#### Neuropsychological Assessment

All participants completed a detailed standardized neuropsychological assessment, which covered multiple cognitive domains. General cognitive function was assessed by the Montreal Cognitive Assessment test (MoCA) (32). Attention was assessed by the Trail Making Test (TMT A and B) (33). One Back Test (OBT) (34) and digit span task (from MoCA) were used to identify the working memory performance. Geriatric Depression Scale (GDS scale) (35) was used to rule out older participants who are suffering from depression. Further, both the groups were from similar socioeconomic background and were matched on education level. In addition, performance on general cognitive assessment (MoCA) was equivalent across groups, indicating similar cognitive ability (Refer to Table 1).

**Table 1:** Demographic, neuropsychological, and language measures of both the groups.

	**Young adult (*N* = 20)**	**Older adult (*N* = 18)**	***t***	**Sig. (2-tailed)**
	**Mean (SE)**	**Mean (SE)**		
**Demographic information**				
Age	32.6 (0.7)	73.9 (0.6)	−41.6	0.00^*^
Education	18.7 (0.7)	16.8 (0.6)	1.8	0.087
Gender	Female = 9	Female = 11		
**Neuropsychological assessments**				
MoCA	29.2 (0.1)	28.61 (0.2)	1.9	0.095
TMT A	16.8 (0.7)	30.09 (1.9)	−6.4	0.00^*^
TMT B	39.7 (2.3)	60.02 (5.3)	−3.5	0.00^*^
OBT_RT	751.3 (34.1)	931.5 (40.7)	−3.4	0.00^*^
OBT_Acc	0.9 (0.007)	0.8 (0.01)	1.7	0.098
**Subjective measures of LP**				
L2: Percent exposure	26.5 (3.4)	18.3 (2.8)	1.7	0.08
L2: AoA-Speaking	7.4 (0.7)	8.3 (0.7)	−0.9	0.36
L2: AoA-Reading	10.7 (0.9)	12.9 (1.1)	−1.4	0.15
L2: LP-Speaking (Max:10)	7.3 (0.3)	6.2 (0.4)	1.8	0.07
L2: LP-Reading (Max:10)	7.9 (0.3)	7.2 (0.3)	1.6	0.12
**Objective scores on the measures of LP**				
L2 LexTale (%)	80.5 (2.3)	81.9 (2.1)	−0.4	0.66
L2 BNT (Max:60)	48.8 (1.4)	46.2 (1.3)	1.3	0.19
L2 RC (Max:11)	5.9 (0.4)	6.2 (0.43)	−0.5	0.60
L2 Discourse (Max:18)	17.02 (0.1)	16.7 (0.3)	0.7	0.43
L2 Composite LP scores (%)	77.5 (0.01)	77.2 (0.01)	0.1	0.89

SE, Standard error; MoCA, Montreal Cognitive Assessment; TMT, Trial Making Test; OBT, One back Test; RT, Response time; Acc, Accuracy; LP, Language Proficiency; L2, Second Language; AoA, Age of Acquisition; BNT, Boston Naming Test; RC, Reading Comprehension;

* *, significant*.

#### Measures of Bilingualism

*Second* language (L2) age of acquisition (AoA), language usage and proficiency were established by the Language Experience and Proficiency Questionnaire (LEAP-Q) (36), a widely used subjective measures of bilingualism (Refer to Table 1). LEAP-Q was used to collect information on the L2 speaking and reading AoA, percentage of L2 usage in daily life in speaking and reading domains, as well as self-reported L2 speaking and reading proficiency (Refer to Table 1). Four objective measures of L2 proficiency were also included (a) L2 proficiency in confrontation naming (Boston Naming Test) (37), (b) L2 proficiency in discourse production^1^ using pictures from Western Aphasia Battery (41) and Boston Diagnostic Aphasia Examination (42) that provides a composite rubric score (38, 39), (c) L2 proficiency in reading comprehension using a part of York adult assessment battery- revised [YAA-R; (43)], and (d) L2 proficiency in vocabulary skills using LexTale test (44).

#### Attention Network Test (ANT)

**Figure 1:**
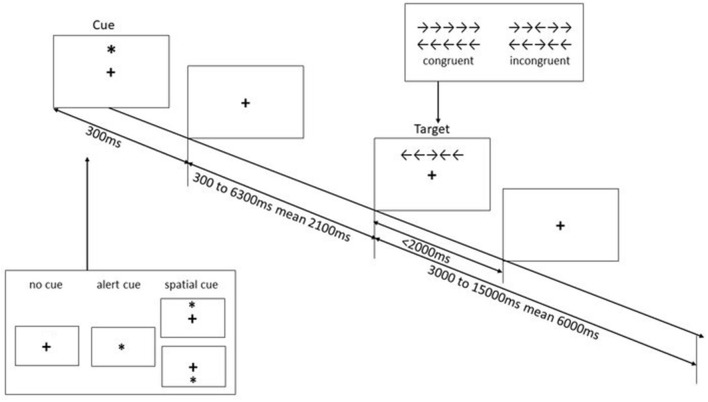
Schematic of attention network test (1). This figure illustrates the time course of the warning cues and the flanker condition.

We used event-related fMRI to study the activations of the different subcomponents of attention. This task is a combination of the cueing paradigm (45) and the flanker task (46). Participants were presented with five white arrows on a black background and were asked to determine the direction of the target arrow in the middle—left or right. The arrows were presented either above or below a centrally located fixation cross. The target arrow was flanked by pairs of congruent arrows or incongruent arrows, resulting in two flanker conditions—congruent and incongruent, respectively. Furthermore, three types of warning cues were used: no-cue (baseline), alert cue (temporally informative), and spatial-cue (temporally and spatially informative). The efficiency of the three attentional effects was assessed by measuring how response times are influenced by different warning cues and flanker conditions resulting in alerting (No cue vs. alert cue), orienting (Spatial cue vs. alert cue) and executive control (Incongruent vs. congruent flanker condition) effects. Each trial begins with a fixation window, followed by the cue window lasting for 300 ms. After a variable duration (one of a set of 12 discrete times from 300 to 6,300 ms, approximating an exponential distribution with a mean interval of 2,100 ms), the stimuli appear either above or below the fixation point (based on the warning cue) within two degrees of visual angle until the participant responded or 2,000 ms elapsed. The duration between the onset of the target and the start of the next trial was also jittered (a set of 12 discrete times from 3,000 to 15,000 ms, approximating an exponential distribution with a mean of 6,000 ms; refer to Figure 1). A total of 144 trials (72 for each flanker conditions, namely congruent and incongruent) with different warning cues were recorded in three runs. Each run consists of a 2-buffer trial at the beginning of the run, which was not included in the analysis.

#### Image Acquisition and Processing

MR imaging was carried out using 3T MRI Siemens Prisma Fit scanner with a standard 64-channel head coil. The image sequence was a T2 weighted pulse sequence (TR = 785 ms; TE = 30 ms; matrix = 64 × 64 voxels; FOV = 192 mm; flip angle = 54°; slice thickness = 3 mm; and acquisition = 39 slices). A high-resolution structural image was obtained before the three functional runs using a 3D T1-weighted imaging using an MPRAGE sequence (TR = 2,400 ms; TE = 2.33 ms; 240 slices; matrix = 256 × 256 mm; voxel size = 0.8 × 0.8 × 0.8 mm; and FOV = 230 mm). Imaging data were pre-processed and analyzed using SPM12 (Wellcome Department of Imaging Neuroscience, UK) running with MATLAB (Mathworks, USA). To correct for between-scan movements, the functional images were realigned to the first image of each session. Each participants' structural T1 image was then co-registered to the mean functional image. The functional images were then spatially normalized into the standard space defined by the Montreal Neurological Institute (MNI) template. After normalization, all scans were resampled at a resolution of 2 × 2 × 2 mm. The functional images were spatially smoothed with an isotropic Gaussian kernel (full width at half maximum of 8 mm) to increase the signal-to-noise ratio.

### Statistical Analysis

#### Behavioral Data Analysis

Raw response time, accuracy rate and inverse transformed response time (RTinv = −1,000/RT) were used for the data analysis. Inverse transformation was performed to normalize the positively skewed response time distribution. Mixed ANOVA and ANCOVA were performed with groups (OA vs. YA) as between-subject variable and flanker conditions (congruent vs. incongruent) and warning cues (no, alert, or spatial) as the within-subject variables for the accuracy and RTinv data (with average response time of each participant as covariate), respectively. The overall slowing of stimulus perception and motor response for the older adults have confounding effect of age (47) in the behavioral measures of ANT performance. To address this concern in the response time analyses, ANCOVAs using average response time as a covariate was conducted. Also, incorrect responses and the trials with response time exceeding three standard deviations were excluded from the analysis. Ratio scores were subsequently computed for the alerting effect [(No cue–Alert cue)/Alert cue], orienting [(Alert cue–Spatial cue)/Spatial cue] and the executive control effect [(Incongruent–Congruent)/Congruent] using the raw response time. Furthermore, these scores were introduced for a planned comparison between the groups using independent sample *t*-test. Instead of the conventional subtraction measure (1, 12), ratio scores were used to define the efficiency of the subcomponents of attention, thus, reducing the influence of the overall response time (RT) on the alerting, orienting and executive control effects (48, 49).

#### fMRI Data Analysis

The general linear model (GLM) in SPM was used to conduct a whole-brain analysis of the fMRI data. We created a design matrix using the onset time of the events (separate events for no, alert, spatial cues, and congruent and incongruent flanker conditions with correct responses only). Incorrect responses and the buffer trials of each run were combined as an extra column in the design matrix. These events were convolved with the canonical hemodynamic response function (HRF), and the six motion correction parameters for each functional run were included in the design matrix as covariates of no interest. The regressors were fitted to the fMRI data to produce beta estimates for each regressor. Individual subject and second level (random effects) group analyses were conducted. Contrasts were same as the behavioral analysis, except inverted for the alerting and orienting effects (i.e., alerting fMRI effect = alert cue beta estimate – no cue; orient fMRI effect = Spatial cue beta estimate – alert cue). Only effects surviving an uncorrected voxel-level threshold of *P* < 0.001 and/or a cluster level familywise error (FWE) corrected threshold of *P* < 0.05 were interpreted with cluster size of at least 20 voxels. For group-level analyses, the independent sample *t*-test was performed to assess the difference between the young and older adults for each contrast—alerting, orienting and executive control contrast (i.e., executive control fMRI effect = incongruent beta estimate – congruent condition). In absence of any group difference, the one-sample *t*-test was performed to determine group-level activation for that particular contrast. Region-of-interest (ROI) analyses were performed for each of the three contrasts based on the previous study (1), with a priori defined ROIs of 5 mm radius. The percent signal changes within the pre-determined ROIs (1) were calculated using MarsBar toolbox (50) for each of the contrast and were analyzed using the independent sample *t*-test between the groups using SPSS.

#### Correlation Analysis

The relationship between measures of bilingualism (subjective and objective measures of L2 performances) and attention performance (behavioral and neurofunctional) was further examined by doing a Pearson correlation analysis with adjusted *p*-values controlling for multiple comparisons. Also, behavioral and neurofunctional performance were correlated to assess the relationship between activation pattern and the behavioral measures of ANT performance. To do so, composite factor scores for the measures of bilingualism and behavioral performance (Accuracy and response time) was calculated by performing a principal component analysis with varimax rotation method. This allows to reduce the number of correlations and avoids the effect of cross-correlations between different factor scores. In the factor analysis, factor scores were calculated using the Bartlett method in SPSS, which were then used in the correlation analysis.

## Results

### Behavioral Results

**Figure 2:**
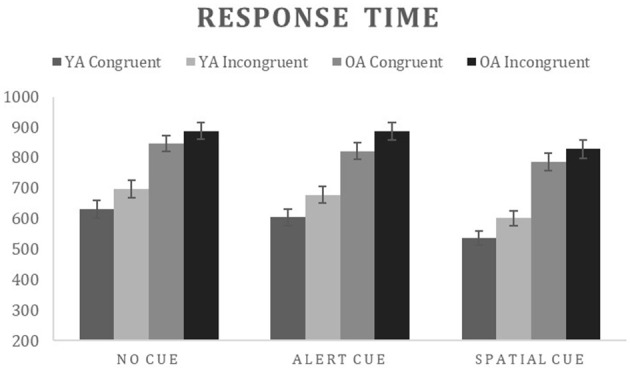
Mean response time and accuracy for each condition on the attention network task. OA, Older adults; YA, Young adults.

Accuracy for the correct trials was submitted to a mixed ANOVA with group as a between subject variable (YA and OA) and warning cue (no, alert, spatial) and flanker condition (congruent, incongruent) as within subject variables. The main effects of warning cue [F_(2, 72)_ = 41.06, $\eta_{p}^{2}$ = 0.533, *p* < 0.000], and flanker condition [F_(1, 36)_ = 54.54, $\eta_{p}^{2}$ = 0.602, *p* < 0.000] were significant. The only significant interaction was between warning cue and flanker condition [F_(2, 72)_ = 36.57, $\eta_{p}^{2}$ = 0.504, *p* < 0.000] indicating that the flanker effect (Incongruent > congruent) was present only for the no cue and spatial warning cues (Table 2 for details). RTinv for correct trials were submitted to a 2^*^3^*^2 ANCOVA with group as a between subject variable (OA and YA) and warning cue (no, alert, spatial) and flanker condition (congruent, incongruent) as within-subject factors, with average response time of each participant as covariate (Figure 2). Response times varied as a function of warning cue [F_(2, 70)_ = 9.82, $\eta_{p}^{2}$ = 0.219, *p* < 0.000; no cue > alert cue > spatial cue] and flanker condition [F_(1, 35)_ = 5.204, $\eta_{p}^{2}$ = 0.129, *p* = 0.029; Incongruent > congruent], with significant two way interaction between warning cue and flanker condition [F_(2, 70)_ = 3.58, $\eta_{p}^{2}$ = 0.093, *p* = 0.033] indicating that the flanker effect (Incongruent > congruent) was present for all warning cues, however, alerting effect (No cue > Alert cue) for the incongruent condition was not significant, indicating difficulty in processing the alerting cue for the participants in conflict trials. A significant main effect of group [F_(1, 35)_ = 5.887, $\eta_{p}^{2}$ = 0.144, *p* = 0.021] as well as interaction between group and flanker condition [F_(1, 35)_ = 6.27, $\eta_{p}^{2}$ = 0.152, *p* = 0.017] and a group and warning [F_(2, 70)_ = 7.13, $\eta_{p}^{2}$ = 0.169, *p* = 0.002] were observed. The nature of the interaction between age and warning cue is readily apparent in Figure 2. For Older adults, the magnitude of warning cue effects—alerting (No cue – Alert cue) and orienting effect (Alert cue – Spatial cue)—were smaller when compared to young adults, indicating that with increasing age the ability to take advantage of the warning cues reduces. As for the interaction effect between group and flanker condition, young adults showed the desired flanker effect (Incongruent > Congruent) for all the warning cues, whereas older adults showed flanker effect only for the alerting cue. Thus, resulting in smaller interferences effect for the older adults when compared to young adults.

**Table 2:** Mean RT (and SD) and accuracy (and SD) for each condition during ANT behavioral performance.

**Flanker condition**	**Age group**	**Warning type**		
		**No cue**	**Alert cue**	**Spatial cue**
**Accuracy**				
Congruent	YA	97.5 (0.7)	98.5 (0.7)	98.2 (0.7)
	OA	97.6 (1.1)	97.6 (0.8)	98.5 (0.8)
Incongruent	YA	91.4 (1.8)	91.6 (1.6)	95.2 (1.2)
	OA	93.2 (2.5)	94.2 (1.9)	96 (1.2)
**Response time**				
Congruent	YA	631.11 (74.9)	604.23 (71.3)	536.45 (60.9)
	OA	846.37 (161.2)	821.75 (157.5)	787.12 (139.0)
Incongruent	YA	698.38 (82.2)	678.56 (85.83)	601.77 (91.6)
	OA	887.35 (145.01)	887.42 (152.6)	828.79 (159.74)

*YA, Young Adults; OA, Older Adults*.

A significant three-way interaction for group, flanker condition and warning cue [F_(2, 70)_ = 5.29, $\eta_{p}^{2}$ = 0.131, *p* = 0.007] showed a larger alerting, orienting and executive control effect for the young adults. The effect of the covariate—verage response time—was significant indicating that some of the age-related difference in response time observed for the warning cue and flanker conditions can be attributed to general slowing [F_(2, 70)_ = 3.63, $\eta_{p}^{2}$ = 0.094, *p* = 0.032]. However, the effect of group remained significant after controlling for age-related slowing, suggesting that age-related changes in attentional ability other than general slowing also contributed to differences in response time between younger and older adults. *Post hoc* analysis of the three-way interaction showed significant group differences for the congruent condition for all warning cues (*p* < 0.05) indicating older adults are slower than younger adults on taking advantage from the warning cues on trials without any conflict (i.e., congruent). For the incongruent condition, group difference was evident only for the spatial cue (OA > YA; *p* < 0.05) indicating that in the conflict condition (i.e., incongruent), older adults were slower to take advantage from the temporally and spatially informative cue (i.e., spatial cue). And the planned comparison of the ratio scores for the three subcomponent of attention revealed that older adults (relative to young adults) showed a numerically smaller alerting [*t*_(36)_ = 2.13, *p* = 0.03], orienting effect [*t*_(36)_ = 3.58, *p* = 0.001] as well as significantly smaller interference effect [i.e., executive control effect; *t*_(36)_ = 2.68, *p* = 0.01].

### fMRI Results

**Figure 3:**
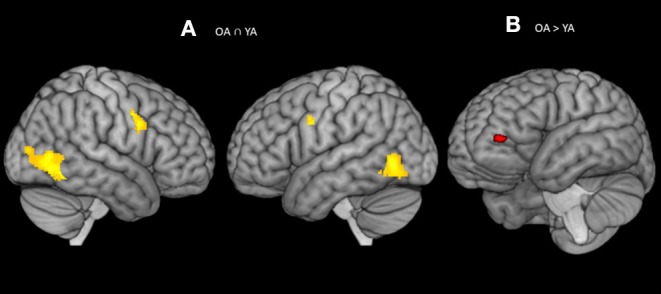
**(A)** Significant blood-oxygen-level dependent (BOLD) signal increase related to the alerting contrast (Alert cue – No cue) in both the groups together (OA n YA) revealed bilateral activation in the fusiform gyrus (BA 37) and pre-SMA (BA 6). **(B)** Significant blood-oxygen-level dependent (BOLD) signal increase related to the alerting contrast in the Older bilinguals in comparison to young bilinguals (OA > YA) revealed activation in the ventrolateral PFC (BA 10). Statistical parametric maps overlaid on the average T1-weighted anatomy of all subjects (*p* < 0.001 and *K* > 20).

**Figure 4:**
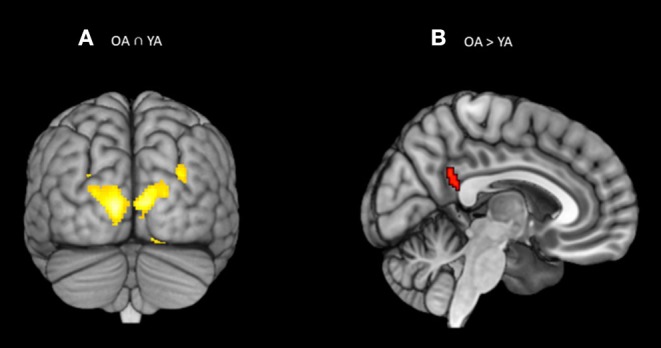
**(A)** Significant blood-oxygen-level dependent (BOLD) signal increase related to the orienting contrast (Spatial cue – alert cue) in both the groups together (OA n YA) revealed bilateral activation in the visual association areas (BA 18). **(B)** Significant blood-oxygen-level dependent (BOLD) signal increase related to the orienting contrast in the Older bilinguals in comparison to young bilinguals (OA > YA) revealed activation in the superior parietal gyrus (BA 39). Statistical parametric maps overlaid on the average T1-weighted anatomy of all subjects (*p* < 0.001 and *K* > 20).

**Figure 5:**
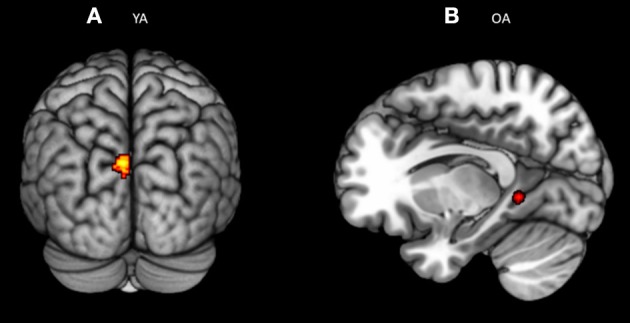
Significant blood-oxygen-level dependent (BOLD) signal for **(A)** young bilinguals and **(B)** older bilinguals for the executive control contrast (Incongruent – Congruent). Statistical parametric maps overlaid on the average T1-weighted anatomy of all subjects (*p* < 0.001 and *K* > 20).

Firstly, we identified common brain regions that were consistently engaged in young and older adults for each subcomponent of attention in a random effect analyses (see Table 3). Secondly, two-sample *t*-test was performed on contrast images to assess the significance of any group differences observed in the activation patterns between young and older adults for different subcomponents of attention (see Table 3; Figures 3–5).

**Table 3:** Results from the random-effects analyses for the alerting, orienting, and executive control condition, for young and older adults.

**Contrast**	**Anatomical region**	**WB/ROI**	**Area**	**Side**	**MNI coordinates**			**Volume**
					**x**	**y**	**z**	
**Alerting**								
OA∩YA”	Fusiform gyrus	WB	BA 37	Rt	42	−56	−14	899
		WB	BA 37	Lt	−40	−62	−6	703
	Precentral gyrus	WB	BA 6	Lt	−46	2	34	82
		WB	BA 6	Rt	46	4	40	138
OA > YA^*^	VLPFC	WB	BA 10	Lt	−26	50	−10	24
	IFG	ROI	BA 47	Lt	−33	31	−3	
**Orienting**								
OA∩YA”	Visual association area	WB	BA 18	Lt	−10	−98	4	413
		WB	BA 18	Rt	10	−96	8	153
		WB	BA 18	Lt	−20	−78	−10	115
		WB	BA 18	Rt	18	−76	−14	139
OA>YA^*^	Superior parietal gyrus	WB	BA 39	Rt	42	−50	28	29
**Executive control^∧^**								
Young adults		WB	BA 19	Lt	−4	−86	36	28
Older adults	Isthmus of CG	WB	BA 30	Lt	−22	−50	6	20

* *Reverse contrast showed no effect*.

∧ *Conjunction and disjunction analysis did not result in any effect*.

*“Survived cluster level FEW. k > 20. IFG, Inferior frontal gyrus; VLPFC, ventrolateral prefrontal cortex; CG, Cingulate gyrus; WB, Whole Brain; ROI, Region of Interest*.

### Comparison Between Young and Older Adults for the Alerting Effect

The conjunction analysis^2^ on the brain activity associated with the alerting (Older adults n Young adults), defined as increased activity in alert cue trials vs. no cue trials, showed activation in the bilateral fusiform gyrus (BA 37) and pre-Supplementary Motor Area (pre-SMA; BA 6) (see Table 3; Figure 3A). The no and Alert cues trials were collapsed over congruent and incongruent flanker conditions. Disjunction analysis (Older adults > young adults) revealed differential increases in neural activity in the left ventrolateral prefrontal cortex for the older adults (Lt BA 10, *p* = 0.001 uncorrected, *k* > 20) (Figure 3B; Table 3). No significant activation was observed for the reversed contrast. Neural correlates of alerting were also observed in the pre-defined regions-of-interest within the left inferior frontal gyrus [Lt BA 47, defined in Fan et al. (1)]. We did not find any group difference for the rest of the pre-defined ROIs.

### Comparison Between Young and Older Adults for the Orienting Effect

The conjunction analysis on the brain activity associated with the orienting ability (Older adults n Young adults), defined as increased activity in spatial cue trials vs. alert cue trials, showed activation in the bilateral visual association areas (BA 18; see Table 3; Figure 4A). The two-sample *t*-test (Older adults > young adults) revealed differential increases in neural activity in the right superior parietal gyrus close to temporal parietal junction (Rt BA 39, *p* = 0.001 uncorrected, *k* > 20) (Figure 4B; Table 3). The reverse contrast revealed no significant increases in neural activations for young adults relative to older adults. Same as alerting ability, the Spatial and Alert cues were collapsed over congruent and incongruent flanker conditions. No significant activation was found within the a priori defined regions-of-interest for the orienting contrast.

### Comparison Between Young and Older Adults for the Executive Control Effect

Whole brain analysis as well as Region-of-interest analysis did not show any significant neural activation common to both the groups (Older adults n young adults) or distinct between groups (Older adults > < young adults) for the executive control effect, defined as an increase in brain activity in incongruent vs. congruent conditions, by collapsing all the warning cues together. However, we identified brain regions that were consistently engaged in young bilinguals and in older bilinguals, in separate random effects analyses for executive control effect (Incongruent > congruent). Older adults showed activity in the isthmus of left cingulate gyrus [Left BA 30; *p* = 0.001 (uncorrected), *k* > 20] whereas young adults recruited more posterior brain for the same task [Left middle occipital gyrus BA 19 *p* = 0.001 (uncorrected), *k* > 20; Figure 5].

### Relationship Between Neurofunctional Activation and Behavioral Measures

Results from the individual factor analysis of the subjective and objective measures of bilingualism (Refer to Table 1) produced a single and two-factor solution, respectively (refer to Tables 4A,B for details). All the subjective measures of bilingualism—LEAP Q—yielded a single factor structure. The factor analysis (Tables 4A,B) of the objective measures resulted in two-factors: L2 proficiency in discourse production and rest of the objective measures (L2 naming, L2 reading comprehension, and L2 vocabulary). Similarly, ANT behavioral performance resulted in three-factor solution—response time measures, accuracy measure for incongruent flanker condition and accuracy for congruent flanker condition.

**Table 4A:** Combined factor analysis of both the groups for the ANT behavioral performance.

			**ANT behavioral performance**					
	**Factor**	**Variance**	**No congruent**	**Alert congruent**	**Spatial congruent**	**No incongruent**	**Alert incongruent**	**Spatial incongruent**
Factor 1	Response time	49.06%	0.971	0.973	0.969	0.971	0.968	0.964
Factor 2	Accuracy for incongruent conditions	20.15%				0.822	0.846	0.761
Factor 3	Accuracy for congruent conditions	10.90%	0.825	0.595	0.463			

**Table 4B:** Combined factor analysis of both the groups for the measure of bilingualism.

			**Objective measures of bilingualism**				**Subjective measures of bilingualism**						
	**Factor**	**Variance**	**L2 naming**	**L2 vocabulary**	**L2 reading Comprehension**	**L2 discourse production**	**Factor**	**Variance**	**L2 language exposure**	**L2 AoA speaking**	**L2 AoA reading**	**L2 speaking proficiency**	**L2 reading proficiency**
Factor 1	L2 naming, vocabulary and reading comprehension	45.85%	0.919	0.767	0.656	0.95	Single factor	52.87%	0.710	−0.481	−0.660	0.835	0.881
Factor 2	L2 discourse production	27.25%				0.917							

Pearson's correlation analyses were conducted to examine the links between behavioral effects—using the factor scores—and brain activation in the corresponding subcomponent of attention. To test for the relationship between the attentional abilities to more general neuropsychological performance as well as with proxies of cognitive reserve—education and measures of bilingualism—we correlated activation thresholds with the neuropsychological measures, education, and measures of bilingualism. Also performed partial correlations with education as covariate to find the relationship with the measures of bilingualism. We then apply Bonferroni correction for multiple testing to the results. We find a positive correlation between the neural activity of the VLPFC (BA 10) and the composite factor score for the response time (all the flanker conditions and the warning cues (*r* = 0.455; *p* = 0.001), indicating that with an increase in neural activity there is an increase in behavioral response time. There was no significant correlation between the neural activity of the superior parietal region (BA 39) related to orienting ability and the factor scores for accuracy and response time measures However, we find a positive correlation between the response time on the working memory task and the BA 39 activity (*r* = 0.454, *p* = 0.004), indicating increased neural activity of BA 39 with an increase in response time. Factor scores of behavioral ANT performance—i.e., Factor 1 = representing a composite measure of accuracy on congruent condition—showed a positive correlation with the factor scores of L2 proficiency in discourse production across the group (*r* = 0.624, *p* = 0.01) while controlling for education as a covariate. Interestingly, this correlation was significant with the older bilinguals only (OA, *r* = 0.607, *p* = 0.01; YA, *r* = 0.34; *p* = 0.23). Factor scores of behavioral ANT performance (Factor 3 representing composite measure of accuracy on congruent condition) showed positive correlation with the factor scores with Factor 2 of the objective measure of bilingualism (L2 proficiency in discourse production*; r* = 0.624, *p* = 0.01) and more so for older bilinguals (*r* = 0.607, *p* = 0.01), while controlling for education as covariate. BOLD activity for the VLPFC (BA 10)—indicating an increase in neural activity for the alerting ability in elderly—showed negative correlation with Factor 2 of the objective measure of bilingualism (L2 proficiency in discourse production*; r* = −0.517, *p* = 0.001) across group; with the older bilinguals there was an increase in this correlation value (*r* = −0.655) indicating that with an increase in L2 proficiency in discourse production there is a decrease in BOLD activity for the region related to alerting ability. For the alerting contrast (OA > YA) no significant correlation was seen with education level. Also, partial correlation continues to give similar results while controlling for education (covariate), BOLD activity of VLPFC continue to show a negative correlation with L2 proficiency in discourse production (*r* = −0.523, *p* = 0.001). For the orienting contrast (OA > YA), no correlation was seen between the BOLD activity and the proxies of cognitive reserve—education and bilingualism (with and without partial correlation).

## Discussion

The study intended to describe the fMRI brain activation patterns associated with the subcomponents of attention in young and elderly bilinguals, and to look for the association with the measures of bilingualism—L2 age, usage, and proficiency. Two groups of participants i.e., young and elderly bilinguals performed the Attention Network task during fMRI scanning. L2 usage and proficiency were looked at as influencing the pattern of activation for the subcomponents of attention in young and old bilinguals. Both the groups—young and elderly bilinguals—were matched on the factors of education level, and L2 usage and proficiency, as well as for neuropsychological variables and L2 usage and proficiency were normally distributed within each group, thus making them continuous variables. Response times (RTinv), accuracy rates, and BOLD activation on flanker conditions—congruent and incongruent—and warning cues—no, alert and spatial cues—were computed, to examine alerting, orienting, and executive control effects in young and older bilinguals. As a whole, neurofunctional and behavioral results show that alerting and orienting abilities are significantly lower in elderly bilinguals as compared to young bilinguals, a finding that is associated with an increase in neural activity in elderly bilinguals, particularly in the fronto-parietal complex, sub-serving top-down attention control processes.

More specifically, both age groups were equivalent in terms of accuracy of responses. Conversely, significant differences in response times across groups show that the elderly bilinguals do not benefit from warning cues as much as the young do, and get more distracted by flankers, particularly when there is a spatial cue. These findings replicate previous findings with the ANT, showing larger executive control effects and smaller alerting effects, in older adults as compared to younger adults (30, 51–53). Also, in line with the results of previous behavioral studies showing age-related decline specific to the executive control ability only (31). Previous studies with bilingual population converge with the notion that a bilingual advantage is seen in executive control when compared to monolingual groups (12, 23, 24, 54). In the present study, the age-related differences were not evident in the incongruent condition as the experimental groups were balanced for L2 age of acquisition, language usage, and proficiency, and that could express a lack of differences in conflict resolution in the incongruent condition that requires the use of executive control mechanism. However, it is possible that these behavioral studies (12, 23, 24) are based on the performance of young monolingual and bilinguals, therefore, we must be careful in drawing parallels with the present study where young and older bilinguals varying in L2 usage and proficiency are compared. In sum, behavioral results of the present study confirm previous findings with the ANT, that the early subcomponents of attention—alerting and orienting ability—are a sensitive indicator of age-related differences in L2 matched older and younger adults.

The fMRI results add an important perspective on group differences between young and older bilinguals in the subcomponents of attention. The current study showed an increase in the neurofunctional activation for the alerting and orienting subcomponents of attention for older bilinguals when compared to young bilinguals using disjunction analysis. Specifically, there was a significant activation in the left ventrolateral prefrontal cortex (BA 10) for the elderly bilinguals with alerting trials and a significant activation in the right superior parietal gyrus (BA 39) with the orienting trials. Furthermore, in a region-of-interest analyses for the different subcomponents of attention [predefined areas based on Fan et al. (1)], older adults showed reduced neural activity in the left inferior frontal gyrus (BA 47) with alerting trials, but no significant group difference with orienting and executive control trails in the anterior cingulate, other parietal sites and frontal eye fields.

Neurofunctional patterns in older adults fit well with the previous literature on aging (55, 56), showing that fronto-parietal activity increases with age. This age-related increase in activation was observed concurrently with higher response times on the ANT and working memory tasks (OBT). Specifically, in the elderly, response times to alerting trials on the ANT task were positively correlated with activations of the VLPFC (BA 10). According Cabeza et al. (57), older adults' performance is influenced by an increase in age-related PFC activation, thus confirming the present results. However, we find positive correlation between the response time on the working memory task and the BA 39 activity (*r* = 0.454, *p* = 0.004) indicating increase in working memory performance correlated with reciprocal increase in BA 39 activity. In addition, superior parietal area (BA 39) is reported in literature to play a critical role in covert and overt shift of attention (58) and thereby having a crucial role in attentional shift in space (59). This age-related increase in neural activation in the frontal and parietal areas responsible for the alerting ability also showed corresponding increase in response time for the ANT task performance. Hence, by relating behavioral and BOLD signal changes in the alerting ability, the present work shows that—in comparison to young bilinguals—older bilinguals rely upon the prefrontal cortex (BA 10) to sustain the level of alertness required for the ANT task performance. Also, the positive correlation between response time on the working memory task (One back task) and the activation of the superior parietal gyrus (BA 39) indicates that working memory processing contributes to orienting attention in space. This is in line with previous work showing the role of working memory processes in spatial attention (60). The novelty of the present results relies on the fact that the result shows a correlation between reduced BOLD response in older bilinguals and response times with the alerting and orienting ability, instead of executive control (49, 61). This suggests that age-related differences in the cue-driven performance in the alerting and orienting ability could result from reduced neural efficiency. However, age groups did not differ in regard to the neurofunctional and behavioral patterns of executive control ability, showing significant activations in the left cingulate gyrus (BA 30) and the left middle occipital gyrus (BA 19) for older and young bilinguals, respectively. The lack of result in the disjunction analysis (OA > YA) for the executive control ability can be explained by the fact that both the groups were strictly matched on L2 age of acquisition, usage, and proficiency. This is in line with the previous study (13) that shows comparable neurofunctional activation for the older bilinguals in comparison to the young adults (monolinguals and bilinguals) in executive control ability. Also, the results for young bilinguals on the executive control performance are in line with bilingual anterior to posterior and subcortical shift hypothesis (BAPSS) (62), that suggest less activation of the frontal brain areas responsible for executive function and greater recruitment of posterior/subcortical regions by bilinguals when compared to monolinguals.

We also explored the possibility that decreased neural efficiency in older bilinguals may vary with L2 variables resulting in cognitive reserve. The results show that neural efficiency—decrease in neural activity—is correlated with increasing L2 proficiency as measured by discourse tasks, thus suggesting that higher L2 proficiency through life-long use of the two languages, contributes to neural efficiency for the alerting ability. Research on bilingualism has mostly focused on comparisons between monolingual and bilingual populations, showing both cognitive and neural advantages in bilinguals (8, 18, 63), accounted by neuroanatomical (17, 64–66) and neurofunctional changes (11, 13–15). The present study is the first one to report on age-related differences on behavioral and neurofunctional patterns of attention in comparable bilingual populations differing in age and varying in L2 usage, and proficiency.

In sum, the evidence showed an increase in the brain activity for the older bilinguals in comparison to young bilinguals in the frontal and parietal areas during alerting and orienting subcomponents of attention and this is correlated with lower L2 proficiency and higher working memory response time across group. According to Wang and Fan (67), alerting ability results in broad sensitivity toward incoming stimuli and this ability reduces with increasing age. In the present study, a bilingual advantage in maintaining this alert state is observed in the elderly bilinguals, and this ability is associated with increasing L2 proficiency on discourse tasks. This is in line with the previous studies supporting bilingual advantage in the cognitive control performance on the continuum of L2 proficiency (68, 69). Together, our results suggest that benefits of lifelong bilingualism might rely specifically upon the alerting subcomponent of attention.

## Conclusion

Defining and interpreting age-related differences in bilingual population, based on behavioral and neuroimaging data is an ongoing challenge. In this study, we compared older and younger adults, matched on measures of bilingualism and education, to understand the role of bilingualism in aging. A bilingual advantage was observed, specifically in the alerting ability, a subcomponent of attention responsible for establishing a state of alertness for the incoming stimuli. This finding points to alerting abilities as the potential core component of the so-called bilingual attentional advantage.

## Data Availability Statement

The datasets generated for this study are available on request to the corresponding author.

## Ethics Statement

The studies involving human participants were reviewed and approved by CIUSSS Aging and Neuroimaging Research Ethics Committee of Center-Sud-de-l'Île-de-Montréal. The participants provided their written informed consent to participate in this study.

## Author Contributions

TD carried out the data collection, analysis, and interpretation, and drafted the initial article. TD, AA, and YJ participated in the interpretation, discussion, and manuscript preparation. PB contributed to the fMRI design and analysis of the study. All authors read the final manuscript and approved it for publication.

### Conflict of Interest

The authors declare that the research was conducted in the absence of any commercial or financial relationships that could be construed as a potential conflict of interest.

## Footnotes

^1^L2 discourse scores consist of a composite rubric score (Maximum = 18) that is performed with the help of scoring sheet (38–40), in which total score is calculated by summing the scores on the following aspects: Overall impact and achievement of purpose, Organization and technique, and Mechanics (38–40). Two independent high proficient rates listen to the audio recording and rate the performance on this rubric. Average of the two raters' response is taken as the final score for the L2 proficiency in discourse production.

^2^Conjunction analyses involves identifying brain areas that show significant activation across the experimental condition, in the present study across the group (YAnOA). In contrast, disjunction analyses identify significant activation that is present in one experimental condition and not the other (OA > YA or reverse).
